# Sentinel surveillance of acute respiratory infection (ARI) in children under five years old from 2015 to 2022 at a high-complexity health care institution in Cali, Colombia

**DOI:** 10.7189/jogh.15.04293

**Published:** 2025-10-31

**Authors:** Edilson Iles-Dorado, Johana Alarcón Soto, José Luis Aguirre, Ana Isabel Trochez, María José Trochez, Jenny Elizabeth Ordoñez-Betancourth, Mabel Moreno, Sebastián Romero-Fernández, Paola Marsela Pérez-Camacho, Marly Suleydi Orrego Flórez, José Guillermo Betancourt-Villalobos, Jaime Alberto Restrepo-Tovar

**Affiliations:** 1 Fundación Valle del Lili, Comité de Vigilancia Epidemiológica, Cali, Colombia; 2 Departamento de Salud Pública y Medicina Comunitaria, Facultad de Ciencias de la Salud, Universidad Icesi, Cali, Colombia; 3 Fundación Valle del Lili, Centro de Investigaciones Clínicas, Cali, Colombia; 4 Fundación Valle del Lili, Servicio de Pediatría, Cali, Colombia; 5 Fundación Valle del Lili, Servicio de Infectología Pediátrica, Cali, Colombia

## Abstract

**Background:**

Respiratory viral infections in children under five pose a significant public health challenge worldwide. Sentinel surveillance systems identify viral causes of acute respiratory infections (ARIs), providing real-time data to enhance public health strategies. This study analyses data from 2015–2022 from one of Colombia’s principal sentinel surveillance centres for severe acute respiratory infection (SARI), located in a high-complexity hospital in the Pacific region. While findings reflect a specific local context, they offer valuable insights into viral circulation in a high burden setting and contribute to strengthening national and global surveillance efforts.

**Methods:**

A cross-sectional study was conducted, tracking cases from epidemiological week 1 of 2015 to week 52 of 2022. Viral identification and clinical outcomes were analysed, focusing on intensive care unit (ICU) admissions and associated risk factors.

**Results:**

A total of 3035 cases of respiratory viral infections were analysed from a sentinel hospital between 2015 and 2022. The median age of children was 12 months. Respiratory syncytial virus (RSV) was the most common pathogen (30.9%), followed by influenza (6.7%) and adenovirus (5.1%). Intensive care unit admission rates were higher among children with underlying conditions such as cardiovascular disease and malnutrition (33%). RSV accounted for 33% of ICU cases. Infection rates peaked seasonally, particularly in children under 12 months. Younger age and comorbidities were key risk factors for ICU admission. Conversely, older children and those infected with RSV or influenza had lower ICU admission rates.

**Conclusions:**

Sentinel surveillance data confirm the high burden of RSV and other respiratory viruses in children, particularly those under 12 months and with comorbidities. Findings highlight the need for targeted public health measures, including improved vaccination and health care access for high-risk groups. Strengthening surveillance is essential for timely, evidence-based interventions.

Acute respiratory infection (ARI) is a major public health concern and ranks among the top three causes of morbidity and mortality globally, especially affecting the extremes of life [1,2]. In children, ARIs are the leading cause of infectious disease mortality [2]. In 2019, about one in seven deaths in this age group worldwide were attributed to ARI [3]. Early diagnosis and timely care are essential, yet care-seeking behaviours have declined globally, increasing associated risks [4–7].

Sentinel surveillance systems are key to early detection and mortality reduction. Although the COVID-19 pandemic strengthened these systems, monitoring of other ARIs may have declined during this period. Despite the global burden of COVID-19, other viral ARIs remain major contributors to morbidity and mortality. For example, influenza causes an estimated 39.1 million lower respiratory infections and 58 200 deaths annually, while respiratory syncytial virus (RSV) accounts for 24.8 million cases and 76 600 deaths [8].

Sentinel systems identify viral ARI causes and offer real-time data for public health action. They allow timely interventions to mitigate respiratory infection impacts beyond COVID-19 [9]. Clinical surveillance monitors incidence, severity, and mortality; however, in Colombia, limited published data exists on ARI dynamics across different periods, including pre- and post-pandemic phases [10,11]. Strengthening sentinel surveillance is crucial to improving public health responses and safeguarding health, particularly in resource-limited settings. This study focuses on one of twelve officially designated sentinel sites for SARI in Colombia, consistently ranking first or second in national reporting during the study period [12]. Located in the Pacific region of southwestern Colombia, marked by high disease burden, this centre meets WHO criteria for sentinel surveillance [13].

The Colombian National Institute of Health's (INS) 2022 report showed a sharp rise in health care use compared to 2021: outpatient visits increased by 50.3%, general hospitalisations by 50.8%, and ICU admissions by 41.3% [14]. In children under five, outpatient visits stayed within expected levels early in the year but peaked in September and October. General hospitalisations exceeded historical limits from May, and ICU admissions remained high throughout 2022 [14]. ARI rates reached 9.1 per 100 000 children under five in Valle del Cauca and 4.5 per 1000 live births in Cali, the region’s capital. Although national reports [14] and recent studies from Colombia’s northeast [15] have described overall ARI and virus trends, detailed institution-level analyses from other areas remain scarce. This study addresses that gap by presenting clinical and epidemiological data from a major sentinel hospital in Cali, a densely populated city with high transmission risk. These findings, from systematic tertiary care surveillance, enhance understanding of ARI seasonality and burden in this high-risk setting.

While national surveillance reports (INS, 2023) [16] and studies such as Peña-Buendía et al. (2023) [15] have described general ARI trends in Colombia, institution-level analyses from other regions are scarce. This study presents clinical and epidemiological data on viral respiratory infections in children under five from a high-complexity hospital in Cali, Colombia, a key sentinel site for ILI and SARI surveillance. By describing patient profiles, viral prevalence, and ICU admission associations (2015–2022), the study provides context-specific evidence to guide prevention, care, and surveillance. In low- and middle-income countries, data on the prevalence and sociodemographic profile of sentinel events remain limited. In Colombia, national reports lack disaggregated data for ILI and SARI. Contributions from sentinel institutions are essential to fill these gaps [8,17] and strengthen global SARI surveillance, especially as few countries have established robust systems despite pilot efforts [18].

## METHODS

### Study design

We conducted a cross-sectional study of children under five years reported to the ILI-SARI sentinel surveillance system at a high-complexity hospital in Cali, Colombia. Cases were recorded from epidemiological week 1 of 2015 to week 52 of 2022, following the INS epidemiological weeks [14]. This article is reported in accordance with the STROBE guidelines (Table S8 in the **Online Supplementary Document****).**

According to INS case definition for ILI-ARI (event code 345 in the national health surveillance system), cases were defined as ‘acute respiratory infection with a history of fever or measured fever ≥38°C, cough, onset within the last 10 days, and requiring hospitalisation’ [14]. We included only children residing in Cali to ensure complete sociodemographic and georeferenced data. Patients from other regions were excluded due to low numbers and missing data. This focus enabled consistent characterisation of viral circulation across Cali’s 22 communes.

### Study setting

The study took place at a tertiary hospital in Cali, designated among Colombia’s 12 national SARI sentinel sites [19,20], consistently ranked among the top national in the Colombian SARI surveillance system [12]. By 2024, it operated over 700 beds, including 40 paediatric and 41 neonatal ICU beds, and received over 120 000 emergency visits and 650 000 outpatient consultations annually, serving both urban and rural populations [21].

### Data collection

Sociodemographic and clinical data were obtained from the hospital’s ILI-SARI database, part of Colombia’s national public health surveillance system (SIVIGILA), and managed in Microsoft Excel®. Variables included age, sex, health insurance type (by affiliation regime), and medical history. Socioeconomic status was defined per the Department of Statistics (DANE) urban stratification system, based on housing and environmental conditions [22]. Viral data included test results (positive/negative), identified pathogens and the type of isolation method used. Viral testing followed PAHO recommendations to ensure quality [23].

### Specimen collection and laboratory processing

In accordance with national ARI surveillance protocols, nasopharyngeal aspirates were collected, stored at ≤8°C, and transported to the reference laboratory, then to the INS for confirmatory testing [24]. Pathogen detection used PCR and indirect immunofluorescence, depending on protocols and period [24], including SARS-CoV-2, Influenza A and B, Respiratory Syncytial Virus (RSV), Adenovirus, Parainfluenza viruses 1, 2, and 3, and other respiratory viruses [24]. Hospital tests included STANDARD Q COVID-19 antigen test and BioFire® FilmArray® Respiratory Panel for multiplex detection of respiratory pathogens. From 2015–2022, case definitions, collection procedures, and referral pathways remained consistent. Expanded molecular panels were introduced from 2019, supplementing but not replacing core protocols.

### Data analysis

Data were cleaned following ILI-SARI protocol standards. Exploratory analysis addressed missing values, duplicates, and refined variables such as case definition and hospitalisation status. Population characteristics were described by year (2015–2022). Quantitative variables were summarised using medians and IQRs; categorical variables, as frequencies and percentages. ARI diagnoses were grouped by anatomical site: bronchiolitis, pneumonias, and others (tracheitis, sinusitis). Bivariate comparisons used the Kruskal-Wallis test for non-normally distributed continuous variables and Pearson χ^2^ or Fisher exact test for categorical variables.

Multivariate logistic regression was performed with ICU hospitalisation as the binary outcome. Variables with *P* < 0.25 were initially included [25,26], those with *P* < 0.05 remained in the final model. To explore potential confounding, subgroup analyses were performed by age group (<1 year and 1–5 years) and comorbidity status, using the same outcome. These subgroup analyses also examined annual positivity rates, the distribution of covariates, and the circulation of respiratory agents among ARI patients by hospital ward and ICU admission. Analyses were performed using STATA® version 18.0 (StataCorp LLC, Texas, USA).

## RESULTS

A total of 3035 cases were included in the analysis. The number of cases varied across study years, with the lowest in 2020 (n = 190) and the highest in 2022 (n = 646).

### Sociodemographic and epidemiological characteristics

Most children were affiliated with the contributory health insurance scheme, one of the main regimes within Colombia’s health system, and belonged to socioeconomic strata 2 (low) and 3–4 (middle). This distribution reflects the typical patient population of this high-complexity referral hospital, which primarily admits urban children with chronic or severe conditions. Among reported comorbidities, asthma was the most frequent (5.6%), followed by cardiovascular disease (3.6%) and cancer (3.8%) (**Table 1**).

**Table 1:** Sociodemographic and epidemiological characteristics of patients reported for ARI to the sentinel surveillance system at a high-complexity hospital, Cali, Colombia, 2015–2022

Variable	Total (n = 3035)	2015 (n = 203)	2016 (n = 418)	2017 (n = 392)	2018 (n = 443)	2019 (n = 455)	2020 (n = 190)	2021 (n = 288)	2022 (n = 646)	*P*-value*
**Age in months, MD (IQR)**	12 (7–36)	11 (4–24)	12 (7–24)	12 (5–24)	12 (8–24)	12 (9–36)	12 (8–24)	12 (6–24)	12 (8–36)	0.0001*
**Sex, n (%)**										0.371ƚ
*Male*	1626 (53.6)	100 (49.3)	222 (53.1)	219 (55.9)	218 (49.2)	244 (53.6)	108 (56.8)	157 (54.5)	358 (55.4)	
*Female*	1409 (46.4)	103 (50.7)	196 (46.9)	173 (44.1)	225 (50.8)	211 (46.4)	82 (43.2)	131 (45.5)	288 (44.6)	
**Health insurance scheme, n (%)**										<0.0001‡
*Contributory*	2459 (81)	113 (55.7)	295 (70.6)	331 (84.4)	375 (84.7)	371 (81.5)	153 (80.5)	244 (84.7)	577 (89.3)	
*Subsidized*	273 (9)	28 (13.8)	41 (9.8)	31 (7.9)	36 (8.1)	37 (8.1)	22 (11.6)	32 (11.1)	46 (7.1)	
*Exception*	74 (2.4)	5 (2.5)	12 (2.9)	5 (1.3)	11 (2.5)	26 (5.7)	5 (2.6)	6 (2.1)	4 (0.6)	
*Uninsured*	99 (3.3)	6 (3)	10 (2.4)	9 (2.3)	20 (4.5)	21 (4.6)	9 (4.7)	5 (1.7)	19 (2.9)	
*Special*	121 (4)	51 (25.1)	56 (13.4)	12 (3.1)	1 (0.2)		1 (0.5)			
*No information*	9 (0.3)		4 (0.9)	4 (1)				1 (0.3)		
**Time to consultation in days, MD (IQR)**	3 (2–5)	3 (2–5)	3 (2–5)	3 (2–5)	3 (2–5)	3 (2–5)	3 (1–5)	3 (1.5–4)	3 (2–5)	0.0338*
**Time to notify SIVIGILA in days, MD (IQR)**	2 (1–3)	1 (1–2)	1 (1–2)	1 (1–2)	2 (2–3)	3 (2–4)	1 (1–3)	2 (1–3)	3 (2–4)	0.0001*
**Comorbidity, n (%)**										<0.0001†
*Yes*	969 (31.9)	40 (19.7)	147 (35.2)	130 (33.2)	177 (40)	201 (44.2)	74 (38.9)	39 (13.5)	161 (24.9)	
*No*	2066 (68.1)	163 (80.3)	271 (64.8)	262 (66.8)	266 (60)	254 (55.8)	116 (61.1)	249 (86.5)	485 (75.1)	
**Number of comorbidities, n (%)**										
One										
*Yes*	767 (25.3)	35 (17.2)	119 (28.5)	105 (26.8)	132 (29.8)	139 (30.5)	53 (27.9)	34 (11.8)	150 (23.2)	
*No*	2268 (74.7)	168 (82.8)	299 (71.5)	287 (73.2)	311 (70.2)	316 (69.5)	137 (72.1)	254 (88.2)	496 (76.8)	
Two										
*Yes*	190 (6.3)	4 (2)	26 (6.2)	24 (6.1)	42 (9.5)	58 (12.7)	20 (10.5)	5 (1.7)	11 (1.7)	
*No*	2845 (93.7)	199 (98)	392 (93.8)	368 (93.9)	401 (90.5)	397 (87.3)	170 (89.5)	283 (98.3)	635 (98.3)	
Three or more										
*Yes*	12 (0.4)	1 (0.5)	2 (0.5)	1 (0.3)	3 (0.7)	4 (0.9)	1 (0.5)	0 (0)	0 (0)	
*No*	3023 (99.6)	202 (99.5)	416 (99.5)	391 (99.7)	440 (99.3)	451 (99.1)	189 (9.5)	288 (100)	646 (100)	

ARI – acute respiratory infection, IQR – interquartile range, MD – median

*Kruskal-Wallis test.

†Pearson χ^2^ test.

‡Fisher exact test.

### Distribution of respiratory agents

Of the 3035 cases analysed, RSV was the most frequent pathogen (30.9%), detected in 681 patients in general hospitalisation (30%) and 257 in ICU (33.8%) (**Table 2**).

**Table 2:** Respiratory agents according to hospitalisation or ICU requirement of patients reported for ARI to the sentinel surveillance system at a high-complexity hospital, Cali, Colombia, 2015–2022

Respiratory agents, n (%)	Total (n = 3035)	General hospitalisation (n = 2274)	ICU hospitalisation (n = 761)	*P*-value
RSV	938 (30.9)	681 (30)	257 (33.8)	<0.001
Influenza (All types)	203 (6.7)	173 (7.6)	30 (3.9)	<0.001
Influenza virus	94 (46.3)	82 (47.4)	12 (40)	
*Influenza AH3N2*	38 (18.7)	34 (19.7)	4 (13.3)	
*Influenza AH1N1*	34 (16.7)	29 (16.8)	5 (16.7)	
*Influenza B*	32 (15.8)	25 (14.5)	7 (23.3)	
*Non-typeable Influenza*	5 (2.5)	3 (1.7)	2 (6.7)	
Adenovirus	155 (5.1)	130 (5.7)	25 (3.3)	<0.001
Other respiratory agents*	335 (11.1)	210 (9.2)	125 (16.4)	
Negative test	1404 (46.2)	1080 (47.5)	324 (42.6)	

ARI – acute respiratory infection, ICU – intensive care unit, RSV – respiratory syncytial virus

*Other respiratory agents include: Bocavirus, coronavirus (unspecified), coronavirus 229E, coronavirus HKU1, coronavirus NL63, coronavirus OC43, enterovirus, metapneumovirus, rhinovirus, Streptococcus pneumoniae, Sars CoV-2; others (unspecified).

**Figure 1:**
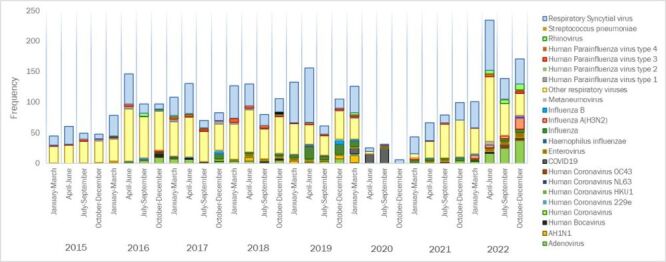
Distribution of respiratory pathogens identified in children reported with acute respiratory infections to the sentinel surveillance system at a high-complexity hospital in Cali, Colombia, 2015–2022.

Influenza virus was found in 203 cases (6.7%), of these, 38 cases were identified as Influenza A H3N2 (18.7%), 34 as Influenza A H1N1 (16.7%), 32 as Influenza B (15.8%), and 5 (2.5%) as non-typeable. Adenovirus was detected in 155 cases (5.1%), showing significant differences (*P* < 0.001). Other respiratory pathogens were identified in 335 patients (11.1%), including Bocavirus, unspecified coronavirus, coronavirus 229e, coronavirus HKU1 and OC43, enterovirus, metapneumovirus, rhinovirus, *Streptococcus pneumoniae* and SARS CoV-2 (COVID-19). A total of 1404 children (46.2%) tested negative for all pathogens. These findings show variation by illness severity. Regarding RSV circulation across the study years, seasonal peaks occurred consistently between April and June during 2015–2018, 2021, and 2022 (**Figure 1**). In contrast, RSV activity was lower and more irregular in 2019 and markedly reduced in 2020, with no clear peak. Influenza and adenovirus showed less consistent seasonality (**Figure 1**). Additional yearly details and breakdowns by hospitalisation status are available in Table S1–5 in the **Online Supplementary Document****.**

Among the 3035 children under five included in the analysis, 19 (0.6%) had received the pneumococcal vaccine (between three and 48 months of age), and 12 (0.4%) the seasonal influenza vaccine (12 to 48 months); no data were available on COVID-19 vaccination. Although vaccination fields are included in the surveillance form (pneumococcal, influenza, and COVID-19 in the version used since 2021), this information was largely missing. Nutritional status was reported for only 23 (0.8%) of 3035 children under five, aged 18 days to 48 months, as having acute malnutrition. Ten (0.3%) children aged 2–60 months died. Due to inconsistent recording, low frequency, and likely underreporting, both variables were excluded from multivariate analyses.

### Positivity rates

Of 3035 ARI cases, 46.3% tested negative for respiratory pathogens – more common in general wards (47.5%) than in ICU (42.6%). Positivity was higher in ICU (57.4%) than in general hospitalisations (52.5%), with significant differences between groups (*P* = 0.001) (Table S1 in the **Online Supplementary Document**).

**Figure 2:**
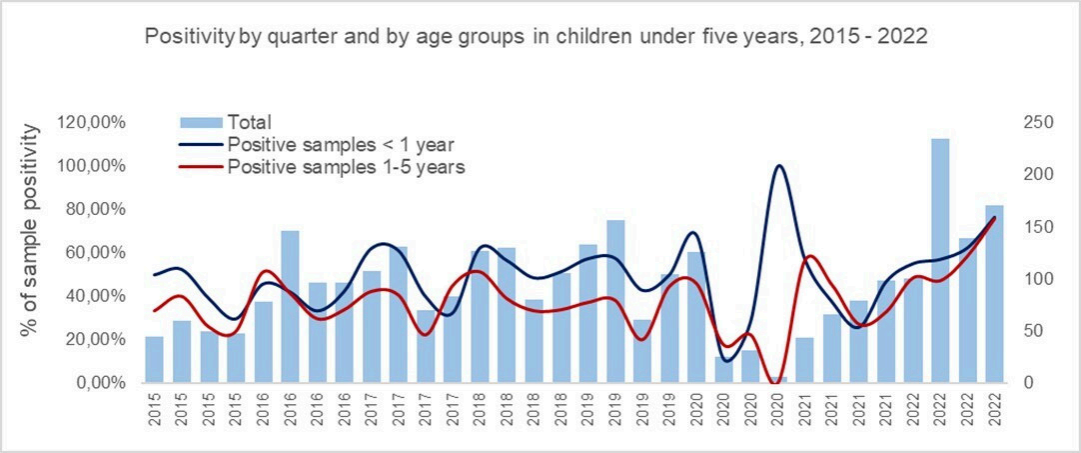
Percentage of sample positivity by trimester and age group in children under five years old, reported for acute respiratory infection (ARI) to the sentinel surveillance system at a high-complexity hospital, Cali, Colombia, 2015–2022.

Analysis of ARI positivity rates by trimester and age group (under 12 months *vs*. 12–60 months) between 2015 and 2022 shows peaks in early 2015, 2018, and 2019, with notable fourth trimester increases in 2017, 2018, and 2022, suggesting seasonal patterns (**Figure 2**). In 2020, rates fluctuated, peaking in the fourth trimester, possibly due to surveillance disruptions. In 2021, positivity rose early, then declined. Rates trended upward again in 2022. Across all years, children under 12 months showed higher and more variable positivity rates, particularly in late 2020 and 2022, indicating possible age-related differences in susceptibility or reporting (**Figure 2**).

### Factors associated with ICU admission

**Table 3** shows odds ratios (ORs) and 95% confidence intervals (CIs) for ICU hospitalisation from unadjusted and adjusted models. The final model included all significant predictors. Comorbidities (CVD, malnutrition, renal insufficiency), pneumonia or bronchiolitis diagnoses, and confirmed respiratory infections increased ICU admission odds (**Table 3**). The strongest predictors were comorbidities: CVD or malnutrition nearly quadrupled the odds of ICU admission, and renal insufficiency increased it more than 5-fold (**Table 3**). However, wide confidence intervals suggest limited precision due to small sample sizes.

**Table 3:** Factors associated with hospitalisation or ICU requirement of patients reported for ARI to the sentinel surveillance system at a high-complexity hospital, Cali, Colombia, 2015–2022

	Total (n = 3035)	General hospitalisation (n = 2274)	ICU hospitalisation (n = 761)	Unadjusted model		Adjusted model	
				**OR (CI 95%)**	***P-*value**	**OR (CI 95%)**	***P*-value**
Age in months, MD (IQR)	12 (7–36)	12 (8–36)	12 (5–24)	0.98 (0.98–0.99)	<0.001	0.98 (0.98–0.99)	<0.001
Comorbidities, n (%)							
*Cardiovascular disease (no CVD as reference)*	118 (28.2)	53 (18.7)	65 (48.1)	3.72 (2.57–5.40)	<0.001	3.31 (2.25–4.88)	<0.001
*Malnutrition (no malnutrition as reference)*	23 (5.5)	10 (3.5)	13 (9.6)	4.16 (1.81–9.52)	0.001	3.64 (1.48–8.95)	0.005
*Renal disease (no renal disease as reference)*	8 (1.9)	3 (1.1)	5 (3.7)	5.29 (1.26–22.19)	0.023	5.70 (1.15–28.12)	0.032
Diagnostics, n (%)							
*Pneumonia*	1306 (43)	985 (43.3)	321 (42.2)	1.95 (1.48–2.56)	<0.001	2.01 (1.52–2.67)	<0.001
*Bronchiolitis*	1186 (39.1)	836 (36.8)	350 (46)	2.45 (1.87–3.23)	<0.001	2.25 (1.68–3.00)	<0.001
*Others (reference)*	543 (17.9)	453 (19.9)	90 (11.8)	1.00	-	1.00	
Respiratory agents, n (%)							
*RSV*	938 (30.9)	681 (30)	257 (33.8)	1.23 (1.01–1.49)	0.033	0.75 (0.52–1.08)	0.125
*Influenza*	203 (6.7)	173 (7.6)	30 (3.9)	0.55 (0.36–0.84)	0.006	0.47 (0.29–0.77)	<0.003
*Adenovirus*	155 (5.1)	130 (5.7)	25 (3.3)	0.57 (0.36–0.92)	0.021	0.42 (0.24–0.76)	<0.004
Other respiratory agents*	335 (11.1)	210 (9.2)	125 (16.4)	1.99 (1.54–2.57)	<0.001	1.39 (0.94–2.06)	0.095
*Negative test (reference)*	1404 (46.2)	1080 (47.5)	324 (42.6)	1.00	-	1.00	
Positivity, n (%)							
*Negative*	1404 (46.3)	1080 (47.5)	324 (42.6)	1.00	-	1.00	
*Positive*	1631 (53.7)	1194 (52.5)	437 (57.4)	1.29 (1.10–1.53)	0.002	1.53 (1.09–2.16)	0.013

ARI – acute respiratory infection, CI – confidence interval, CVD – comorbidities, ICU – intensive care unit, IQR – interquartile range, MD – median, OR – odds ratio, RSV – respiratory syncytial virus

*Bocavirus, coronavirus (unspecified), coronavirus 229E, coronavirus HKU1, coronavirus NL63, coronavirus OC43, enterovirus, metapneumovirus, rhinovirus, Streptococcus pneumoniae, Sars CoV-2; others (unspecified).

In the age-stratified analysis (<1 year and 1–5 years), respiratory viruses showed divergent associations (Table S6 in the **Online Supplementary Document**). For RSV, the overall model suggested a non-significant protective effect (OR = 0.75; 95% CI = 0.52–1.08), which reversed in infants <1 year (OR = 1.30; 95% CI = 0.62–2.73) and became significantly protective in those aged 1–5 years (OR = 0.46; 95% CI = 0.23–0.91). Influenza remained protective: non-significant in <1 year (OR = 0.81; 95% CI = 0.41–1.51), but significant in 1–5 years (OR = 0.55; 95% CI = 0.31–0.98). Adenovirus showed overall protection (OR = 0.42; 95% CI = 0.24–0.76), was neutral in infants (OR = 0.93; 95% CI = 0.70–1.23), and shifted toward non-significant increased risk in older children (OR = 1.23; 95% CI = 0.92–1.65). These unexpected patterns are discussed below.

A similar pattern was observed in models stratified by comorbidities status (Table S7 in the **Online Supplementary Document**). In this subgroup analysis, neither influenza nor adenovirus infection were associated with ICU hospitalisation in any subgroup, while the inverse association between RSV infection and ICU hospitalisation became stronger and statistically significant.

## DISCUSSION

### Summary of results

This study provides evidence on the clinical and epidemiological profile of ARIs among children under five years of age admitted to a high complexity hospital in Cali, Colombia (2015–2022). RSV was the most frequently detected pathogen, but over half of detections involved other viruses. ICU admission was associated with higher positivity and comorbidities – especially cardiovascular disease, malnutrition, and renal insufficiency – while RSV, influenza, and adenovirus infections were linked to lower ICU admission odds.

### Dynamics of acute respiratory infections

In Colombia, the available data on the dynamics of acute respiratory infections (ARI) remain limited and fragmented, with few published studies exploring viral circulation patterns. Early reports from Bogotá described respiratory syncytial virus (RSV) as the leading cause of respiratory illness in children attending a military hospital [27,28]. More recent studies highlighted marked regional differences in RSV transmission between Bogotá and Cali [29], influenced by local climate and viral diversity. To date, only one publication from a sentinel surveillance institution has addressed ARI viral dynamics in Colombia, focusing on a hospital in the northeastern border region [15]. However, this evidence provides only a partial view of national ARI circulation. Our study contributes to filling this gap by providing data from a high complexity hospital and sentinel site in southwest Colombia, applying a more comprehensive analysis of viral circulation and seasonality. It also reflects on the strengths and limitations of existing surveillance systems and their role, and gaps, in informing public health interventions.

We described the sociodemographic profile of children under five with ARI during the study period. Most were covered by the contributory social security system and belonged to lower or middle socioeconomic strata as defined in Colombia. Asthma was the most common comorbidity, and RSV was the predominant pathogen. These findings are consistent with the broader literature, which consistently identifies RSV as a leading cause of paediatric ARI [30–32]. Similar studies have reported high RSV prevalence in hospitalised children, particularly in general wards, while influenza and adenovirus are more common in less severe cases and thus more prevalent in general hospitalisations rather than ICUs [33,34]. This pattern mirrors global trends in respiratory infections and emphasises the need for targeted RSV surveillance and interventions, particularly in socioeconomically disadvantaged populations.

Seasonal patterns and local factors may partly explain the predominance and timing of RSV circulation observed in this study (**Figure 1**). Consistent with previous reports, RSV tends to spread from south to north in the Southern Hemisphere, peaking between March and June [35], often coinciding with the rainy season in tropical areas like Colombia [29,36]. In our data, RSV peaks occurred mainly from April to June, aligning with national rainfall patterns [36]. Colombia's geographic diversity, including variations in altitude and climate, likely contributes to heterogeneous RSV circulation [37,38]. The high proportion of ARI cases linked to pathogens other than RSV, influenza, and adenovirus highlights the complex and context-specific aetiology of respiratory infections in this population [39].

Positivity rate fluctuations from 2015 to 2022 suggest the influence of seasonal patterns, surveillance changes, and external factors such as the COVID-19 pandemic. Higher positivity was observed in the first and last trimesters of several years. In 2020, a sharp peak in the fourth trimester likely reflected reporting disruptions or actual case increases. Children under 12 months consistently showed higher and more variable positivity rates than older children, particularly in late 2020 and 2022. These findings highlight the importance of considering both temporal and demographic factors when interpreting ARI positivity trends [40,41].

Key factors shape ARI surveillance and testing evolution. Effective surveillance ensures adequate case capture and high-quality laboratory samples [13]. The complexity and accuracy of testing have increased with the introduction of PCR panel testing [42], such as the BioFire® FilmArray® Respiratory Panel in use [43], which aims to identify a wide range of pathogens, including bacteria. However, high case volumes can compromise test accuracy. Historically, specific tests for influenza were initially sent to a reference laboratory. However, by 2022, the hospital where this study was conducted transitioned to using PCR panels for these tests. This shift from targeted tests to more comprehensive testing methods aims to improve better handling of suspected cases. Samples were collected and sent to the regional reference laboratory, and results were adjusted in the hospital data set as needed. This adjustment process highlights the importance of maintaining high standards in sample handling and result reporting.

While the sentinel surveillance case definition and sample referral procedures remained constant between 2015 and 2022, molecular diagnostics improved, notably with PCR panel use and specific RSV tests. These advances likely enhanced detection and contributed to higher positivity rates in later years, as shown in the main results and in the **Online Supplementary Document**. However, they also introduce variability across the study period, a common challenge in long-term surveillance [9].

### Factors influencing ICU admission in paediatric ARI

The multivariate model offers insight into factors influencing ICU admission among children with ARI. Comorbidities were key determinants of disease severity and ICU need, with cardiovascular disease, malnutrition, and renal insufficiency particularly associated with increased risk. Children with CVD, for example, had nearly 4-fold greater odds of ICU admission, consistent with previous findings on the vulnerability of children with chronic conditions to severe ARIs [44,45]. Subgroup analysis revealed that, in the presence of comorbidities, the relative contribution of acute clinical factors to ICU admission appears attenuated. This suggests that the baseline risk is already elevated in these patients, likely lowering the threshold for ICU admission even in the presence of moderate clinical deterioration.

Conversely, older age and infections with influenza or adenovirus were associated with reduced ICU admission odds. Although counterintuitive, this may reflect early intervention and structured care pathways. In our setting, for instance, a positive RSV test formally triggers hospitalisation, often leading to earlier care. Similar mechanisms may apply to children with asthma or other chronic respiratory conditions, who typically receive close monitoring and prompt treatment [46–48].

Stratified analysis by age group (<1 year and 1–5 years) revealed greater complexity. RSV infection was not significantly associated with ICU admission overall but increased risk in infants and was protective in older children, likely reflecting age-specific severity profiles and admission thresholds. Influenza infection consistently showed a protective association, particularly among 1–5-year-olds, potentially due to systematic antiviral use (*e.g*. Oseltamivir) and enhanced clinical monitoring upon diagnosis [47,49]. In contrast, adenovirus showed variable patterns. While the overall model indicated a protective effect, age-stratified results suggested a non-significant increase in ICU risk among older children. This heterogeneity may reflect differences in clinical presentation and limitations of sentinel surveillance data. Notably, adenovirus has been linked to severe outcomes in other studies in Colombia [50], warranting further investigation.

### Limitations of sentinel surveillance for ARIs in resource-constrained settings

By 2022 PCR testing had become more widespread in our study, with 485 PCR tests conducted. Distinguishing between hospital-based and reference-laboratory testing remains important, as testing methods can influence positivity rates and reporting. While PCR is more sensitive and relatively affordable its routine use in global surveillance remains limited [51] despite being essential for identifying pathogens such as COVID-19, RSV, influenza, and adenovirus.

Ideally, surveillance data would include individual-level information on vaccination status, nutrition, and antiviral use [52]. However, current public health systems often lack the operational capacity to systematically capture these variables. In theory, medical records could fill this gap, but adherence to documenting such data are low in routine clinical settings [53]. In Latin America, and Colombia in particular, the lack of integrated electronic health records exacerbates this limitation [54]. Retrospective data collection is inefficient and often beyond the practical scope of institutional surveillance. To improve data completeness, especially for key variables like vaccination and comorbidities, stronger coordination between clinical, epidemiological, and national immunisation programmes is needed. Digital vaccination registries or automated data linkage systems may offer feasible solutions.

In Colombia, ARI sentinel surveillance began in 2012, with institutions selected based on feasibility, sustainability, and population representativeness [19,20]. These sites follow standardised procedures for case detection, sample collection, and reporting to SIVIGILA, ensuring data quality [20]. Although this study was conducted in a single sentinel institution in Cali, one of Colombia’s largest ARI reporting centres [12], located in a high-transmission urban area, the use of data from such sites represents a strength. Thus, while caution is needed when extrapolating these findings to other regions, they offer valuable insights into respiratory virus circulation in comparable settings and support efforts to strengthen public health surveillance.

Future research should address key gaps by:

i) assessing disease severity in children without comorbidities to clarify pathogen-specific impacts;

ii) broadening research to include adults for cross-age comparisons; and

iii) conducting clinical studies on key pathogens (*e.g*. adenovirus) using data sources not affected by the limitations of the current surveillance system, and including sentinel sites without high-complexity units to improve generalisability. These steps are essential to strengthen surveillance and generate more representative evidence to inform public health responses.

## CONCLUSIONS

This study demonstrates the high burden of ARIs among children under five in Colombia. RSV was the most frequently detected virus, and ICU admissions were influenced by seasonal trends, comorbidities, and socioeconomic conditions. Although limited to one sentinel hospital and affected by missing data on vaccination and nutrition, the findings reinforce the importance of strengthening surveillance systems, improving diagnostic capacity, and addressing public health gaps to reduce ARI-related morbidity and mortality in resource-limited settings.

## Additional material


Online Supplementary Document
