# Supplementary material for: Sentinel surveillance of acute respiratory infection (ARI) in children under five years old from 2015 to 2022 at a high-complexity health care institution in Cali, Colombia
Source: J Glob Health. 2025 Oct 31;15:04293. doi: 10.7189/jogh.15.04293 (PMC12576863; doi:10.7189/jogh.15.04293)
Supplement: Online Supplementary Document [file jogh-15-04293-s001.pdf]

Supplement to: Iles-Dorado E, Alarcón-Soto J, Aguirre JL, Trochez AI, Trochez MJ, Ordoñez-Betancourth JE, Moreno M, Romero-Fernández S, Pérez-Camacho PM, Orrego-Flórez MS, Betancourt-Villalobos JG, Restrepo-Tovar JA. Sentinel surveillance of acute respiratory infection (ARI) in children under five years old from 2015 to 2022 at a high-complexity healthcare institution in Cali, Colombia. J Glob Health. 2025;15:04293.

Table S1 in the Online Supplementary Document. Sample positivity according to hospitalization or ICU requirement of patients reported for ARI to the sentinel surveillance system at a high-complexity hospital, Cali, Colombia, 2015-2022

Positivity	Total (n = 3035)	General Hospitalization (n = 2274) n (%)	ICU Hospitalization (n = 761) n (%)	P value
Negative	1404 (46.3)	1080 (47.5)	324 (42.6)	0.001*
Positive	1631 (53.7)	1194 (52.5)	437 (57.4)	

* Pearson's chi-squared test

Table S2 in the Online Supplementary Document. Sociodemographic and epidemiological characteristics according to hospitalization or ICU requirements of patients reported for ARI to the sentinel surveillance system at a high-complexity hospital, Cali, Colombia, 2015-2022

Variable	Total (n= 3035)	Inpatient Hospitalization (n= 2274)	ICU Hospitalization (n= 761)	P value
Age in months. Median (IQR)	12 (7-36)	12 (8-36)	12 (5-24)	<0.001*
Sex. n (%)				
Male	1626 (53.6)	1191 (52.4)	435 (57.2)	0.022ǂ
Female	1409 (46.4)	1083 (47.6)	326 (42.8)	
Health insurance scheme. n (%)				
Contributory	2459 (81)	1888 (83)	571 (75)	
Subsidized	273 (9)	149 (6.6)	124 (16.3)	
Exception	74 (2.4)	42 (1.8)	32 (4.2)	<0.001 ǂ
Uninsured	99 (3.3)	82 (3.6)	17 (2.2)	
Special	121 (4)	104 (4.6)	17 (2.2)	
No information	9 (0.3)	9 (0.4)		
Time to consult (days). Median (IQR)	3 (2 - 5)	3 (2 - 5)	3 (2 - 5)	0.097*
Time to notify SIVIGILA (days). Median (IQR)	2 (1 - 3)	2 (1 - 3)	2 (1 - 3)	0.199*
Comorbidity. N (%)				
Yes	969 (31.9)	653 (28.7)	316 (41.5)	<0.001ǂ
No	2066 (68.1)	1621 (71.3)	445 (58.5)	
Number of comorbidities. N (%)				
One	767 (25.3)	538 (23.7)	229 (30.1)	<0.001ǂ
Two	190 (6.2)	111 (4.9)	79 (10.4)	<0.001ǂ
Three or more	12 (0.4)	4 (0.2)	8 (1)	0.003ǂ
Type of comorbidity. N (%)				
Asma				
Yes	150 (4.9)	122 (5.4)	28 (3.7)	0.063ǂ
No	2885 (95.1)	2152 (94.6)	733 (96.3)	

Cardiac disease				
Yes	118 (3.9)	53 (2.3)	65 (8.5)	<0.001 ǂ
No	2917 (96.1)	2221 (97.7)	696 (91.5)	
Cancer				
Yes	113 (3.7)	93 (4.1)	20 (2.6)	0.065 ǂ
No	2922 (96.3)	2181 (95.9)	741 (97.4)	
Malnutrition				
Yes	23 (0.8)	10 (0.4)	13 (1.7)	0.001 ǂ
No	3012 (99.2)	2264 (99.6)	748 (98.3)	
Renal disease				
Yes	8 (0.3)	3 (0.1)	5 (0.7)	0.027 ǂ
No	3027 (99.7)	2271 (99.9)	756 (99.3)	

*Kruskal-Wallis test. ƚ Pearson's chi-squared test. ǂ Fisher's exact test.

Table S3 in the Online Supplementary Document. Percentage distribution of patients by type of service and final diagnosis according to ICD-10 of patients reported for ARI to the sentinel surveillance system at a high-complexity hospital, Cali, Colombia, 2015-2022

Diagnosis	Total (N= 3035)	Inpatient Hospitalization (N= 2274)	ICU Hospitalization (N= 761)	P value
Pneumonia	1306 (43)	985 (43.3)	321 (42.2)	<0.0001
Bronchiolitis	1186 (39.1)	836 (36.8)	350 (46)	
Rhinopharyngitis	365 (12)	312 (13.7)	53 (7)	
Laryngotracheitis	107 (3.5)	70 (3.1)	37 (4.9)	
Sinusitis	48 (1.6)	48 (2.1)		
Tonsillitis	23 (0.8)	23 (1)		

Table S4 in the Online Supplementary Document. Percentage of sample positivity by year and age group of patients reported for ARI to the sentinel surveillance system at a high-complexity hospital, Cali, Colombia, 2015-2022

Year	Age group (Months)	Positive samples	Total samples collected	% Positivity
General	Under 12 months	590	1144	51,57%
	12 to 60 months	802	1891	42,41%
2015	Under 12 months	44	102	43,14%
	12 to 60 months	31	101	30,69%
2016	Under 12 months	70	170	41,18%
	12 to 60 months	94	248	37,90%
2017	Under 12 months	85	163	52,15%
	12 to 60 months	88	229	38,43%
2018	Under 12 months	98	176	55,68%
	12 to 60 months	107	267	40,07%
2019	Under 12 months	81	149	54,36%
	12 to 60 months	114	306	37,25%
2020	Under 12 months	38	69	55,07%
	12 to 60 months	46	121	38,02%
2021	Under 12 months	45	108	41,67%
	12 to 60 months	67	180	37,22%
2022	Under 12 months	129	207	62,32%
	12 to 60 months	255	439	58,09%

Table S5 in the Online Supplementary Document. Percentage of sample positivity by year, trimester and age group of patients reported for ARI to the sentinel surveillance system at a high-complexity hospital, Cali, Colombia, 2015-2022

Year	Trimester	Under 1 year			1 to 5 years		
		Total positive samples	Total simples collected	% Positivity	Total positive samples	Total simples collected	% Positivity
2015	1	6	12	50,00%	11	33	33,33%
2015	2	21	40	52,50%	8	20	40,00%
2015	3	9	23	39,13%	7	27	25,93%
2015	4	8	27	29,63%	5	21	23,81%
2016	1	16	35	45,71%	22	43	51,16%
2016	2	32	76	42,11%	29	70	41,43%
2016	3	11	33	33,33%	19	64	29,69%
2016	4	11	26	42,31%	24	71	33,80%
2017	1	23	37	62,16%	30	71	42,25%
2017	2	41	67	61,19%	26	64	40,63%
2017	3	10	25	40,00%	10	45	22,22%
2017	4	11	34	32,35%	22	49	44,90%
2018	1	33	53	62,26%	38	74	51,35%
2018	2	30	53	56,60%	30	77	38,96%
2018	3	17	35	48,57%	15	45	33,33%
2018	4	18	35	51,43%	24	71	33,80%
2019	1	27	47	57,45%	32	86	37,21%
2019	2	34	59	57,63%	37	97	38,14%
2019	3	9	21	42,86%	8	40	20,00%
2019	4	11	22	50,00%	37	83	44,58%
2020	1	28	41	68,29%	39	85	45,88%
2020	2	1	9	11,11%	3	17	17,65%
2020	3	4	14	28,57%	4	18	22,22%
2020	4	5	5	100,00%	0	1	0,00%
2021	1	12	21	57,14%	13	23	56,52%
2021	2	9	24	37,50%	19	42	45,24%
2021	3	8	31	25,81%	13	48	27,08%
2021	4	15	32	46,88%	22	67	32,84%
2022	1	16	29	55,17%	35	72	48,61%
2022	2	52	91	57,14%	68	144	47,22%

2022	3	25	40	62,50%	58	99	58,59%
2022	4	36	47	76,60%	94	124	75,81%

Table S6 in the Online Supplementary Document. Subgroup Analysis by Age Groups – Multivariable Logistic Regression Model

Variable	Adjusted Model		
	**General Model** OR [CI 95]	**<1 year** OR [CI 95]	**1-5 years** OR [CI 95]
Age in months. Median (IQR)	0.98 [0.98-0.99]		
**Comorbidities. N (%)**			
- Cardiovascular disease (no CVD as reference)	3.31 [2.25-4.88]	2.47 [1.43-4.24]	4.01 [2.28-7.06]
- Malnutrition (no malnutrition as reference)	3.64 [1.48-8.95]	1.57 [0.51-4.81]	10.35 [1.88-56.89]
- Renal disease (no renal disease as reference)	5.70 [1.15-28.12]	----	5.13 [0.97-27.06]
**Diagnostic. N (%)**			
- Pneumonia	2.91 [1.52-2.67]	2.88 [1.80-4.59]	1.77 [1.23-2.52]
- Bronchiolitis	2.25 [1.68-3.00]	2.21 [1.42-3.41]	2.27 [1.53-3.38]
- Others (reference)	1.00	1.00	
**Respiratory agents. N (%)**			
- RSV	0.75 [0.52-1.08]	1.30 [0.62-2.73]	0.46 [0.23-0.91]
- Influenza	0.47 [0.29-0.77]	0.81 [0.41-1.51]	0.55 [0.31-0.98]
- Adenovirus	0.42 [0.24-0.76]	0.93 [0.70-1.23]	1.23 [0.92-1.65]
- Other respiratory agents	1.39 [0.94-2.06]	1.33 [0.88-2.00]	2.55 [1.80-3.59]
- Negative test (reference)	1.00	1.00	1.00

Table S7 in the Online Supplementary Document. Subgroup Analysis by Presence or Absence of Comorbidities -– Multivariable Logistic Regression Model

Variable	Adjusted Model		
	General Model OR [CI 95]	Comorbidities OR [CI 95]	No-comorbidities OR [CI 95]
Age in months. Median (IQR)	0.98 [0.98-0.99]	0.98 [0.97-0.98]	0.97 [0.96-0.98]
**Comorbidities. N (%)**			
- Cardiovascular disease (no CVD as reference)	3.31 [2.25-4.88]	----	----
- Malnutrition (no malnutrition as reference)	3.64 [1.48-8.95]	----	----
- Renal disease (no renal disease as reference)	5.70 [1.15-28.12]	----	----
**Diagnostic. N (%)**			
- Pneumonia	2.91 [1.52-2.67]	1.44 [0.96-2.16]	2.89 [1.93-4.33]
- Bronchiolitis	2.25 [1.68-3.00]	1.89 [1.24-2.88]	2.59 [1.72-3.89]
- Others (reference)	1.00	1.00	1.00
**Respiratory agents. N (%)**			
- RSV	0.75 [0.52-1.08]	0.27 [0.08-0.92]	0.88 [0.52-1.48]
- Influenza	0.47 [0.29-0.77]	0.63 [0.33-1.21]	0.58 [0.33-1.04]
- Adenovirus	0.42 [0.24-0.76]	1.20 [0.85-1.70]	1.03 [0.80-1.32]
- Other respiratory agents	1.39 [0.94-2.06]	2.32 [1.55-3.46]	1.71 [1.19-2.44]
- Negative test (reference)	1.00	1.00	1.00

Table S8 in the **Online Supplementary Document: Strobe checklist for cross sectional studies**

	Item No	Recommendation	Page No
**Title and abstract**	1	(*a*) Indicate the study's design with a commonly used term in the title or the abstract	2
		(*b*) Provide in the abstract an informative and balanced summary of what was done and what was found	2
**Introduction**			
Background/rationale	2	Explain the scientific background and rationale for the investigation being reported	3
Objectives	3	State specific objectives, including any prespecified hypotheses	4
**Methods**			
Study design	4	Present key elements of study design early in the paper	4
Setting	5	Describe the setting, locations, and relevant dates, including periods of recruitment, exposure, follow-up, and data collection	4
Participants	6	(*a*) *Cohort study*—Give the eligibility criteria, and the sources and methods of selection of participants. Describe methods of follow-up<br>*Case-control study*—Give the eligibility criteria, and the sources and methods of case ascertainment and control selection. Give the rationale for the choice of cases and controls<br>*Cross-sectional study*—Give the eligibility criteria, and the sources and methods of selection of participants	5
		(*b*) *Cohort study*—For matched studies, give matching criteria and number of exposed and unexposed<br>*Case-control study*—For matched studies, give matching criteria and the number of controls per case	
Variables	7	Clearly define all outcomes, exposures, predictors, potential confounders, and effect modifiers. Give diagnostic criteria, if applicable	5

Data sources/ measurement	8*	For each variable of interest, give sources of data and details of methods of assessment (measurement). Describe comparability of assessment methods if there is more than one group	*5*
Bias	9	Describe any efforts to address potential sources of bias	5
Study size	10	Explain how the study size was arrived at	
Quantitative variables	11	Explain how quantitative variables were handled in the analyses. If applicable, describe which groupings were chosen and why	5
Statistical methods	12	(*a*) Describe all statistical methods, including those used to control for confounding	5
		(*b*) Describe any methods used to examine subgroups and interactions	5
		(*c*) Explain how missing data were addressed	-
		(*d*) *Cohort study*—If applicable, explain how loss to follow-up was addressed<br>*Case-control study*—If applicable, explain how matching of cases and controls was addressed<br>*Cross-sectional study*—If applicable, describe analytical methods taking account of sampling strategy	5
		(*e*) Describe any sensitivity analyses	-
